# Salivary Aldehyde Dehydrogenases in Oral Toxicology: Biological Functions, Disease Associations, and Translational Perspective

**DOI:** 10.3390/biology15120928

**Published:** 2026-06-14

**Authors:** Masood Alam Khan, Hina Younus

**Affiliations:** 1 Department of Basic Health Sciences, College of Applied Medical Sciences, Qassim University, Buraydah 51412, Saudi Arabia; 2 Interdisciplinary Biotechnology Unit, Faculty of Life Sciences, Aligarh Muslim University, Aligarh 202002, India; hinayounus@rediffmail.com

**Keywords:** salivary ALDH, oxidative stress, oral health, natural compounds

## Abstract

Salivary aldehyde dehydrogenases (ALDHs), particularly ALDH3A1, are increasingly recognized as potential contributors to oral defense against aldehyde-associated stress at the oral–environment interface. Unlike freely secreted salivary enzymes, measurable salivary ALDH activity primarily reflects intracellular and vesicle-associated enzymes derived from salivary gland epithelial cells, oral mucosal cells, immune cells, and exfoliated cellular components. Within the oral exposome, ALDHs expressed in oral epithelial and salivary gland tissues participate in the detoxification of reactive aldehydes, while salivary ALDH activity may serve as an indicator of local aldehyde-detoxification capacity and tissue redox status. Beyond aldehyde metabolism, emerging evidence suggests that ALDH-associated pathways are linked to redox regulation, epithelial stress adaptation, inflammatory signaling, and tissue repair through NAD(P)^+^-dependent processes and stress-responsive networks such as Nrf2 and SIRT1. This review provides a saliva-focused synthesis of ALDH biology, emphasizing isoform-specific functions and the potential importance of ALDH3A1 in oral epithelial defense. Altered salivary ALDH activity has been reported in association with oral conditions including periodontitis, oral lichen planus, radiation-induced salivary dysfunction, and oral squamous cell carcinoma (OSCC). Genetic factors, particularly ALDH2 polymorphisms, together with environmental exposures and microbial dysbiosis, may further influence aldehyde burden and oral disease susceptibility. Although current evidence supports the biological relevance of salivary ALDHs, their utility as clinical biomarkers or therapeutic targets remains investigational and requires further mechanistic and clinical validation.

## Simple Summary

The oral cavity is regularly exposed to reactive aldehydes generated from alcohol consumption, tobacco smoke, oral microorganisms, inflammation, metabolic processes, and environmental exposures. These compounds can contribute to oxidative stress and tissue injury when present at elevated levels. Aldehyde dehydrogenases (ALDHs) are enzymes involved in the metabolism of aldehydes and may help limit their accumulation in oral tissues. This review examines current knowledge of salivary ALDHs, particularly ALDH3A1, and discusses their potential roles in aldehyde metabolism, redox regulation, and oral tissue homeostasis. Altered salivary ALDH activity has been reported in association with several oral conditions, including periodontitis, oral lichen planus, radiation-induced salivary dysfunction, and oral squamous cell carcinoma. While salivary ALDHs may have potential as biomarkers of oral stress and disease, further saliva-specific mechanistic studies and clinical validation are required.

## 1. Introduction

Salivary aldehyde dehydrogenases (ALDHs) are increasingly recognized as critical components of the oral defense system and important molecular links between oral and systemic health [1]. Unlike freely secreted extracellular enzymes, measurable salivary ALDH activity primarily originates from intracellular enzymes released into whole saliva from salivary gland epithelial cells, oral mucosal cells, immune cells, and extracellular vesicles. These enzymes belong to the evolutionarily conserved ALDH superfamily, which catalyzes the NAD(P)^+^-dependent oxidation of endogenous and exogenous aldehydes into less toxic carboxylic acids, thereby limiting aldehyde-induced cellular injury and maintaining redox homeostasis [2,3].

The oral cavity represents a major interface between the human body and the external environment and is continuously exposed to reactive aldehydes generated from alcohol consumption, tobacco smoke, oral microbial metabolism, dietary compounds, environmental pollutants, lipid peroxidation, hyperglycemia, and chronic inflammation [4]. Collectively, these exposures constitute the oral exposome, a dynamic network of endogenous and environmental stressors capable of generating toxic aldehydes such as acetaldehyde, malondialdehyde (MDA), 4-hydroxy-2-nonenal (4-HNE), acrolein, and formaldehyde. In contrast to the broader term oral environment, the oral exposome encompasses the cumulative effects of endogenous and exogenous exposures, including chemical, microbial, dietary, behavioral, and environmental factors that influence oral health. This systems-level framework highlights how diverse aldehyde-generating stressors converge on common pathways of oxidative stress, inflammation, epithelial injury, and carcinogenesis, with salivary ALDHs acting as adaptive protective responders. Tobacco smoke contains numerous reactive carbonyl compounds that induce oxidative DNA damage, protein carbonylation, and epithelial injury, while alcohol exposure substantially increases salivary acetaldehyde levels through microbial and mucosal ethanol metabolism [5]. Oral microorganisms further contribute to aldehyde burden by metabolizing ethanol and carbohydrates into reactive carbonyl species (RCS) within saliva and dental biofilms. In addition, endogenous aldehydes generated through lipid peroxidation and inflammatory oxidative stress amplify epithelial damage and redox imbalance. If inadequately detoxified, these reactive aldehydes form DNA and protein adducts that promote oxidative stress, mutagenesis, chronic inflammation, epithelial barrier disruption, and carcinogenesis [6].

Within the oral exposome, ALDHs expressed in oral epithelial and salivary gland tissues are thought to help limit aldehyde accumulation and protect against chemical stress, while measurable salivary ALDH activity may serve as a non-invasive indicator of these tissue-level protective processes. Among the salivary isoforms, ALDH3A1 is highly enriched in oral and other environmentally exposed epithelial tissues and is increasingly recognized as a potentially important contributor to oral aldehyde metabolism [7]. In contrast, ALDH1A1 participates more broadly in intracellular aldehyde metabolism and retinoic acid biosynthesis, linking it to epithelial differentiation and tissue repair, whereas ALDH2 plays a predominant systemic role in acetaldehyde clearance [8,9]. Individuals carrying the ALDH2*2 polymorphism exhibit impaired acetaldehyde metabolism and an increased risk of upper aerodigestive tract cancers following alcohol exposure [10]. Under such conditions, local ALDH3A1-mediated detoxification within oral tissues may assume greater importance, although direct salivary mechanistic evidence supporting this compensatory role remains limited.

Accumulating evidence links altered salivary ALDH activity with oral diseases, including OSCC, periodontitis, mucositis, oral lichen planus, and diabetes-associated oxidative stress [11,12]. Reduced detoxification capacity may enhance aldehyde-mediated epithelial injury and inflammatory signaling, whereas compensatory ALDH upregulation may reflect adaptive responses to oxidative stress. Salivary ALDH activity is also influenced by oral microbiota composition, aging, hygiene status, metabolic disease, and environmental exposures [13]. Because saliva directly reflects local biochemical and inflammatory changes, salivary ALDHs are emerging as promising non-invasive biomarkers of aldehyde burden, epithelial stress, and oral disease susceptibility. The toxicological relevance of salivary ALDHs is particularly important in OSCC, one of the most common malignancies of the oral cavity. Tobacco products and alcohol exposure introduce substantial aldehyde and carcinogen loads into saliva, including acetaldehyde and numerous International Agency for Research on Cancer (IARC) Group 1 carcinogens [14]. Acetaldehyde generated during ethanol metabolism has itself been classified as a human carcinogen and plays a central role in alcohol-associated oral carcinogenesis [5]. Failure of local aldehyde detoxification mechanisms may therefore contribute directly to chronic epithelial injury and malignant transformation within the oral cavity.

Although ALDH biology has been extensively studied in hepatic and systemic contexts, salivary ALDHs remain comparatively underexplored as distinct components of oral toxicology and mucosal defense. This review focuses specifically on saliva-associated ALDHs, particularly ALDH3A1, as proposed toxicological sentinels of the oral exposome. We discuss their isoform-specific functions, molecular and redox regulatory mechanisms, roles in oral and systemic disease, and emerging translational applications as biomarkers and therapeutic targets. By integrating toxicology, redox biology, oral pathology, and precision therapeutics, this review positions salivary ALDHs as key regulators of oral resilience and disease susceptibility within the oral–systemic health axis.

Several recent reviews have examined ALDH biology, alcohol-associated acetaldehyde toxicity, and aldehyde-mediated oral carcinogenesis [5,9]. However, these studies have primarily focused on systemic ALDH function, alcohol metabolism, or specific disease processes rather than saliva-associated ALDHs and their relevance to oral health. In contrast, this review provides a saliva-focused perspective, examining salivary ALDHs as indicators of tissue-level aldehyde detoxification capacity and potential contributors to oral defense mechanisms. We further apply an oral exposome framework that considers the cumulative impact of alcohol, tobacco smoke, oral microbiota, dietary factors, environmental toxicants, inflammation, and metabolic dysfunction on oral aldehyde burden. By integrating current evidence on salivary ALDH biology, oral exposome-associated stress, and emerging translational applications, this review addresses important knowledge gaps regarding the mechanistic roles, biomarker potential, and therapeutic relevance of salivary ALDHs in oral health and disease.

## 2. Literature Search and Selection

**Figure 1:**
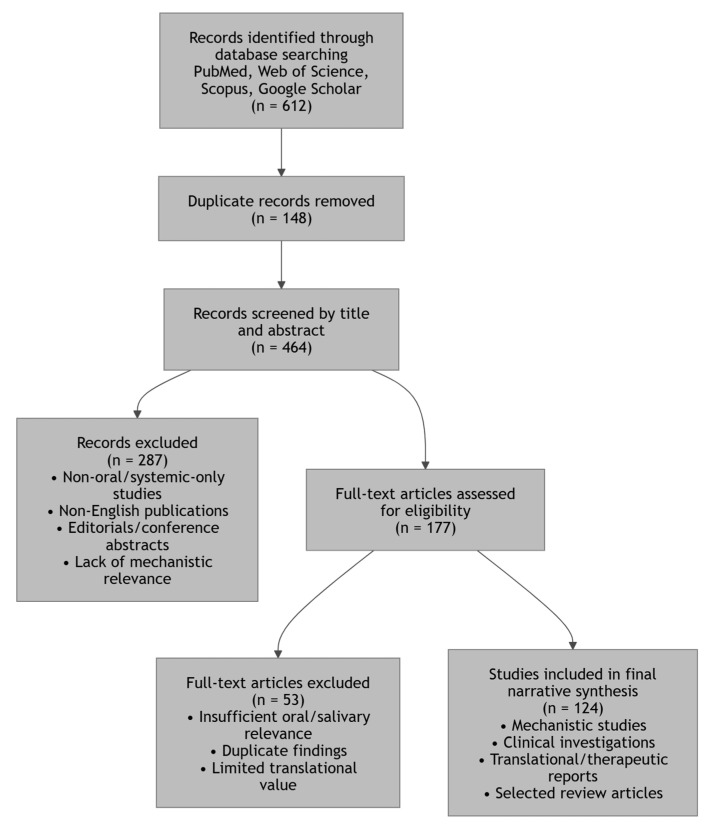
PRISMA-style flow diagram of the literature identification and selection process. The diagram summarizes database searching, duplicate removal, title/abstract screening, eligibility assessment, and final inclusion of studies used in this narrative review on salivary aldehyde dehydrogenases and oral toxicology.

This study was conducted as a narrative review informed by PRISMA-based literature identification and reporting principles to enhance transparency and reproducibility, although no formal systematic review, meta-analysis, or quantitative synthesis was performed (Figure 1). A narrative approach was selected because the available literature on salivary ALDHs is relatively limited and highly heterogeneous, encompassing biochemical, toxicological, experimental, translational, and clinical studies that are not readily amenable to quantitative synthesis. The primary objective was to integrate current knowledge on salivary ALDH biology, oral toxicology, disease associations, and translational applications while identifying key knowledge gaps and future research priorities. A comprehensive literature search was conducted by using PubMed, Web of Science, Scopus, and Google Scholar for studies published between January 2000 and June 2025. Searches combined Medical Subject Headings (MeSH) and free-text terms, including “salivary aldehyde dehydrogenase,” “ALDH,” “ALDH3A1,” “ALDH2,” “oral cavity,” “oral exposome,” “acetaldehyde,” “oral cancer,” “oral squamous cell carcinoma,” “periodontitis,” “oxidative stress,” “oral microbiome,” “ALDH polymorphism,” and “oral–systemic health.” The initial search identified approximately 612 records. After removal of duplicates (n = 148), 464 titles and abstracts were screened for relevance (Figure 1). Of these, 287 records were excluded because they focused exclusively on systemic ALDH biology without oral relevance, lacked mechanistic or clinical relevance to salivary/oral ALDHs, were conference abstracts, editorials, non-English publications, or were unavailable in full text. The remaining 177 articles underwent full-text evaluation, resulting in the inclusion of 124 studies comprising original research articles, clinical investigations, mechanistic studies, and selected review articles directly relevant to salivary or oral tissue-associated ALDH biology.

Inclusion criteria encompassed studies addressing: (i) salivary or oral tissue ALDH isoforms and activity; (ii) aldehyde metabolism and detoxification within the oral cavity; (iii) ALDH-associated oxidative stress, redox regulation, and inflammatory pathways; (iv) oral diseases including oral squamous cell carcinoma, periodontitis, mucositis, and oral potentially malignant disorders; and (v) genetic, environmental, microbial, exposome-related, or therapeutic regulation of ALDH function. Studies were excluded if they lacked direct relevance to oral or salivary ALDH biology, provided insufficient mechanistic or translational insight, represented duplicate reports, or focused exclusively on non-mammalian systems without applicability to oral health.

Because this review was not designed as a formal systematic review, duplicate independent screening, formal risk-of-bias assessment, and quantitative study quality scoring were not performed. Consequently, the potential influence of publication bias, selection bias, and variability in study quality cannot be fully excluded. To address this limitation, evidence derived from direct salivary studies was distinguished from findings obtained in oral epithelial, animal, or systemic ALDH models, and mechanistic inferences were explicitly identified throughout the manuscript where salivary-specific validation is lacking.

## 3. ALDH Superfamily and Isoform-Specific Roles in the Oral Exposome

The aldehyde dehydrogenase (ALDH) superfamily comprises a highly conserved group of NAD(P)^+^-dependent enzymes that protect cells against toxic aldehyde accumulation and maintain redox homeostasis [2]. These enzymes oxidize a broad range of endogenous and exogenous aldehydes into less reactive carboxylic acids, thereby limiting oxidative injury, protein adduct formation, lipid peroxidation, and DNA damage. Among the 19 human ALDH isoenzymes, the ALDH1, ALDH2, and ALDH3 families are particularly important in epithelial defense and aldehyde detoxification.

ALDH1A family enzymes are predominantly cytosolic and catalyze the conversion of retinaldehyde to retinoic acid, linking them to epithelial differentiation, immune regulation, and tissue homeostasis [15]. In contrast, mitochondrial ALDH2 is specialized for systemic detoxification of acetaldehyde generated during ethanol metabolism as well as reactive aldehydes produced during lipid peroxidation [16]. ALDH2 therefore serves as a major regulator of mitochondrial redox balance and aldehyde clearance in metabolically active tissues. ALDH3 isoforms, particularly ALDH3A1, exhibit a distinct epithelial distribution pattern and are highly enriched in tissues exposed to environmental and oxidative stress, including the cornea, respiratory tract, and oral mucosa [17].

Within the oral cavity, ALDH1A1 and ALDH3A1 perform distinct but complementary functions. ALDH1A1 is expressed at relatively lower levels in salivary tissues and contributes primarily to intracellular aldehyde metabolism and retinoic acid-associated signaling pathways involved in epithelial maintenance. By comparison, ALDH3A1 is the predominant salivary epithelial isoform and is proposed to play a major role in epithelial defense against reactive aldehydes generated within the oral exposome. These aldehydes arise from alcohol metabolism, tobacco smoke, microbial activity, dietary compounds, environmental pollutants, lipid peroxidation, and chronic inflammation. ALDH3A1 preferentially detoxifies highly reactive lipid peroxidation-derived aldehydes such as 4-HNE and acrolein, thereby preserving epithelial integrity, limiting oxidative stress propagation, and supporting mucosal resilience.

Experimental studies further suggest that ALDH3A1 contributes to epithelial stress adaptation and tissue regeneration, including protection of salivary stem/progenitor cells following radiation exposure. Unlike systemic ALDH2, which primarily regulates whole-body acetaldehyde metabolism, ALDH3A1 is expressed within oral epithelial and salivary gland tissues that are continuously exposed to aldehydes derived from alcohol, tobacco smoke, microbial metabolism, inflammation, and oxidative stress. This strategic localization suggests a key role in maintaining epithelial resilience under chronic chemical challenge. Supporting this concept, activation of ALDH3A1 in salivary stem/progenitor cells (SSPCs) reduces aldehyde accumulation, enhances cell survival and regenerative capacity, and preserves salivary gland function following radiation exposure without compromising anticancer efficacy [18]. Collectively, these findings indicate that ALDH3A1 contributes not only to aldehyde detoxification but also to epithelial regeneration and adaptive stress responses. However, much of the current evidence is derived from epithelial and experimental models, and further saliva-specific mechanistic studies are needed to validate its proposed role as a major toxicological responder within the oral exposome.

Beyond detoxification, ALDH enzymes participate in broader physiological and pathological processes, including redox signaling, metabolic regulation, neurotransmitter metabolism, lipid turnover, and cellular stress adaptation [19,20]. Dysregulated ALDH expression has been implicated in cancer progression, chemoresistance, inflammatory disorders, and neurodegenerative disease [21]. Elevated ALDH activity is frequently associated with cancer stem cell populations that contribute to tumor aggressiveness and therapeutic resistance [22], whereas ALDH deficiency has been linked to Parkinson’s disease, Alzheimer’s disease, and cardiovascular pathology [23,24].

Clinically, the toxicological significance of ALDH dysfunction is exemplified by the ALDH2*2 polymorphism, which markedly impairs acetaldehyde metabolism after alcohol consumption [25]. Individuals carrying this variant exhibit increased aldehyde accumulation and substantially higher susceptibility to oral and esophageal cancers, inflammatory injury, and oxidative tissue damage. In the context of the oral exposome, impaired aldehyde detoxification may intensify chronic epithelial stress and further increase reliance on local salivary ALDH defense systems, particularly ALDH3A1. Collectively, these findings support the emerging concept that salivary ALDH isoforms, especially ALDH3A1, function not merely as metabolic enzymes but as dynamic regulators of epithelial resilience and toxicological adaptation within the oral cavity.

Although ALDH3A1 exhibits several characteristics consistent with a major oral defense enzyme, including epithelial enrichment and broad aldehyde substrate specificity, its relative contribution to salivary aldehyde detoxification has not yet been quantified in dedicated salivary mechanistic studies.

## 4. Biochemical and Toxicological Basis of Salivary ALDH Function Within the Oral Exposome

**Figure 2:**
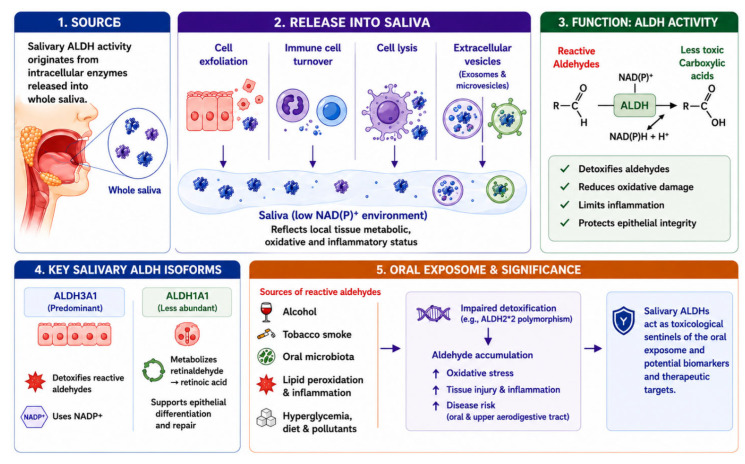
Salivary ALDHs as proposed toxicological sentinels of the oral exposome. Established evidence indicates that measurable salivary ALDH activity primarily reflects intracellular and vesicle-associated enzymes. The figure illustrates mechanistically inferred concepts, including salivary ALDHs as adaptive responders to oral exposome-associated stress and the potential consequences of impaired aldehyde detoxification. These elements represent hypotheses derived from available epithelial and ALDH literature and require further salivary-specific validation.

Salivary ALDH activity primarily originates from intracellular enzymes and may retain enzymatic activity together with associated cofactors (Figure 2). Because extracellular fluids are relatively deficient in NAD(P)^+^ cofactors, measurable salivary ALDH activity should not be interpreted as freely functioning extracellular catalysis, but rather as a dynamic reflection of the metabolic, oxidative, and inflammatory status of local oral tissues. This distinction is particularly important when considering salivary ALDHs as biomarkers of epithelial injury, aldehyde burden, and oral redox imbalance.

Functionally, ALDH enzymes catalyze the NAD(P)^+^-dependent oxidation of reactive aldehydes into less toxic carboxylic acids (Figure 2) [2,26]. Through this detoxification process, ALDHs limit aldehyde-induced DNA damage, protein adduct formation, lipid peroxidation, and inflammatory signaling, thereby contributing to the maintenance of oral mucosal integrity [4]. Structurally, ALDH isoforms share conserved catalytic and nucleotide-binding domains together with oligomerization interfaces essential for enzymatic stability and substrate specificity [19,27]. However, isoform-dependent differences in cofactor preference, tissue distribution, and aldehyde selectivity determine their distinct biological functions within the oral cavity.

The distinct biological functions of salivary ALDH isoforms reflect differences in tissue distribution, substrate selectivity, and metabolic roles [28,29,30,31]. Detailed isoform-specific characteristics are discussed in Section 3. These functional distinctions support the hypothesis that ALDH3A1 may play a prominent role in responding to aldehyde stress within the oral exposome, although direct evidence defining its relative contribution in saliva remains limited, whereas ALDH1A1 contributes more broadly to epithelial metabolic homeostasis.

The oral cavity is exposed to a diverse spectrum of reactive aldehydes originating from alcohol consumption, tobacco smoke, microbial metabolism, lipid peroxidation, hyperglycemia, dietary compounds, and environmental pollutants. Among these, acetaldehyde is particularly important because it is generated locally through ethanol metabolism by oral microorganisms and mucosal enzymes and has been classified as a Group 1 human carcinogen. Beyond forming mutagenic DNA adducts, acetaldehyde impairs DNA repair mechanisms, depletes glutathione reserves, disrupts redox homeostasis, and activates pro-inflammatory signaling pathways that contribute to oral carcinogenesis and mucosal injury [5,9]. Tobacco smoke further introduces highly reactive aldehydes such as acrolein and formaldehyde, which induce protein carbonylation, oxidative stress, mitochondrial dysfunction, and epithelial damage.

Endogenously generated lipid peroxidation products, particularly MDA and 4-HNE, accumulate during oxidative stress and chronic inflammation. These aldehydes readily form protein and DNA adducts, alter cellular signaling pathways, and amplify inflammatory responses. ALDH3A1 appears especially important in detoxifying lipid peroxidation-derived aldehydes within oral epithelial tissues, thereby limiting oxidative injury and maintaining epithelial integrity. In contrast, methylglyoxal, a highly reactive dicarbonyl compound associated with hyperglycemia and advanced glycation end-product (AGE) formation, is detoxified primarily through the glyoxalase system (GLO1/GLO2), while contributions from ALDH isoforms appear secondary and context dependent. Collectively, these aldehydes represent key mediators of oral toxicological stress, highlighting the importance of coordinated detoxification systems, including ALDHs, in preserving oral tissue homeostasis and resilience.

**Figure 3:**
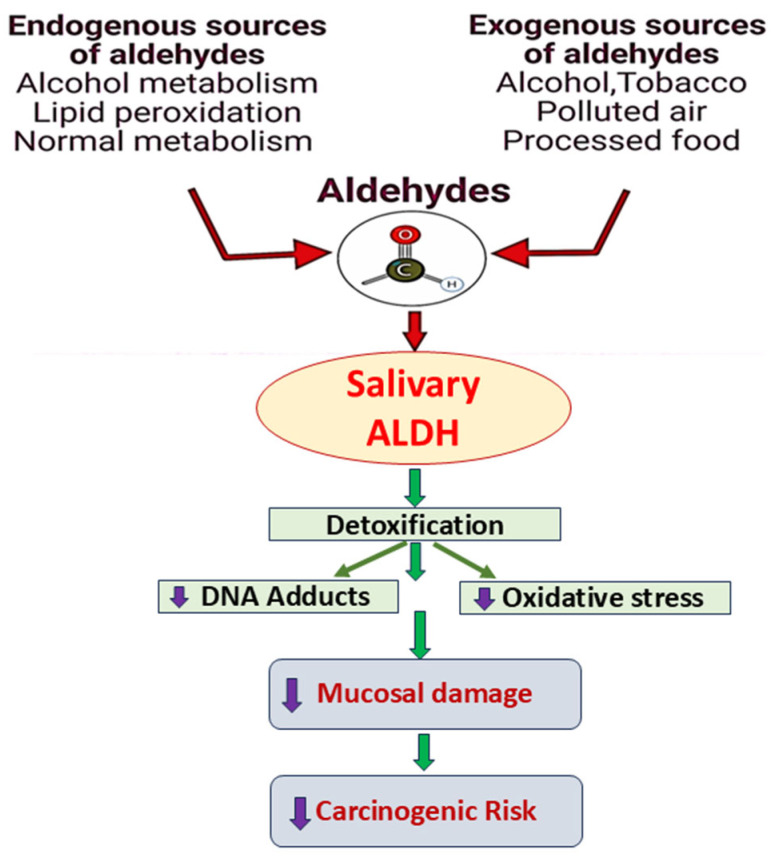
Role of salivary ALDH in aldehyde detoxification. ALDH enzymes associated with oral epithelial and salivary gland tissues catalyze the oxidation of reactive aldehydes into less toxic carboxylic acids. Measurable salivary ALDH activity primarily reflects the aldehyde-detoxification capacity of these tissues and their redox status. Effective aldehyde clearance may limit DNA adduct formation, oxidative stress, and inflammatory injury, thereby helping to preserve mucosal integrity and reduce susceptibility to carcinogenic transformation. The downstream protective effects shown represent literature-supported and mechanistically inferred consequences of aldehyde detoxification rather than direct measurements of salivary enzyme activity.

The oral cavity is continuously challenged by reactive aldehydes generated from alcohol consumption, tobacco exposure, oral microbiota, hyperglycemia, dietary compounds, and chronic inflammation. Within this chemically dynamic environment, salivary ALDHs function as biochemical sentinels that adaptively regulate aldehyde burden and epithelial resilience. In individuals carrying the ALDH2*2 polymorphism, impaired systemic acetaldehyde clearance further increases reliance on local salivary detoxification systems, particularly ALDH3A1 [25]. Under these conditions, inefficient aldehyde metabolism promotes acetaldehyde accumulation and increases susceptibility to oxidative damage and carcinogenesis within the oral and upper aerodigestive tract (Figure 3).

Beyond direct detoxification, salivary ALDHs contribute to broader redox and stress-response networks by limiting the accumulation of aldehydes generated during oxidative stress, lipid peroxidation, and microbial metabolism [32]. This protective activity helps preserve epithelial barrier integrity, suppress inflammatory signaling, and maintain oral tissue homeostasis under chronic chemical stress [33]. Collectively, these observations support the hypothesis that salivary ALDHs, particularly ALDH3A1, may function as proposed toxicological sentinels of the oral exposome, although direct saliva-specific validation remains limited. Throughout this review, references to the protective functions of salivary ALDHs primarily refer to the biological activity of ALDH enzymes within oral epithelial cells, salivary gland cells, and extracellular vesicle-associated compartments that contribute to measurable salivary activity. Saliva is therefore considered principally as a diagnostic matrix reflecting local tissue detoxification capacity rather than the primary site of extensive freely soluble ALDH catalysis.

## 5. Functional Roles of Salivary ALDH in Oral Toxicological Defense

**Figure 4:**
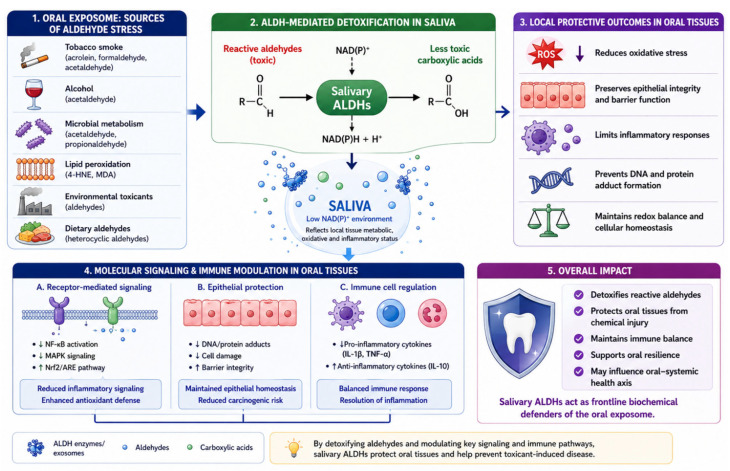
Functional roles of salivary ALDHs in oral toxicological defense. This figure summarizes the principal protective effects associated with ALDH-mediated aldehyde detoxification in oral tissues, including reduction of aldehyde burden, oxidative stress, and epithelial injury. The figure also depicts mechanistically inferred concepts derived primarily from epithelial and broader ALDH studies, including potential interactions with redox-sensitive signaling pathways, inflammatory responses, and immune regulation. These proposed mechanisms have not been fully validated in salivary systems and are presented as hypotheses for future investigation. Collectively, current evidence suggests that salivary ALDH activity may contribute to oral tissue resilience under conditions of aldehyde and oxidative stress within the oral exposome.

Salivary ALDH activity reflects tissue-level aldehyde detoxification capacity and contributes to protection against aldehyde-associated oral toxicological stress (Figure 4) [5]. Through NAD(P)^+^-dependent oxidation of these aldehydes into less reactive carboxylic acids, salivary ALDHs limit oxidative DNA damage, protein adduct formation, epithelial injury, and inflammatory signaling, thereby preserving oral mucosal integrity and redox homeostasis.

### 5.1. Detoxification of Acetaldehyde and Reactive Aldehydes

Among reactive aldehydes present within the oral cavity, acetaldehyde is particularly important because it is classified as a Group 1 carcinogen by the International Agency for Research on Cancer (IARC) and readily forms mutagenic DNA adducts in epithelial tissues [34]. ALDH3A1 expressed in oral epithelial and salivary gland tissues may serve as a frontline detoxification barrier against locally generated acetaldehyde. This protective role becomes especially important in individuals carrying the ALDH2*2 polymorphism, where impaired systemic acetaldehyde metabolism increases dependence on local oral epithelial aldehyde-detoxification mechanisms [35]. Altered salivary ALDH activity has been reported in association with oxidative stress, chronic inflammation, and oral diseases, including oral potentially malignant disorders and oral squamous cell carcinoma [11]. However, the nature and direction of these relationships remain incompletely understood, and current evidence is largely observational. Experimental studies in epithelial models further suggest that ALDH3A1 may help protect against cigarette smoke extract-induced DNA damage and aldehyde-mediated cytotoxicity, supporting a potential role in cellular adaptation to chronic oxidative stress [7].

### 5.2. Redox Homeostasis, Epithelial Protection, and Oral Exposome Adaptation

ALDHs expressed in oral epithelial and salivary gland tissues contribute substantially to cellular defense against oxidative and carbonyl stress, while measurable salivary ALDH activity reflects the status of these protective pathways [36]. Unlike classical antioxidant enzymes that directly neutralize reactive oxygen species (ROS), ALDHs limit the propagation of oxidative injury by metabolizing reactive aldehydes generated during lipid peroxidation, inflammation, microbial metabolism, and environmental exposures. Among salivary isoforms, ALDH3A1 appears particularly important because of its high expression in epithelial tissues and its ability to detoxify lipid peroxidation-derived aldehydes such as 4-HNE, MDA, and acrolein, thereby reducing their cytotoxic, genotoxic, and pro-inflammatory effects [37]. Through its preferential utilization of NADP^+^, ALDH3A1 may also contribute to intracellular redox buffering and support endogenous antioxidant defense systems.

Beyond aldehyde detoxification, ALDH3A1 is increasingly recognized as a contributor to epithelial resilience under conditions of chronic chemical stress. Experimental epithelial studies demonstrate that ALDH3A1 expression attenuates aldehyde-induced cytotoxicity, reduces DNA damage, and enhances resistance to cigarette smoke extract and oxidative injury [7]. These protective effects may support epithelial resilience under chronic aldehyde stress. Consequently, ALDH3A1 has been proposed as an adaptive responder to oral exposome-associated exposures, although this role remains to be validated in salivary systems. Environmental toxicants may further influence ALDH-mediated protection. For example, arsenic exposure has been reported to inhibit human salivary ALDH activity, reducing aldehyde detoxification capacity and potentially increasing susceptibility to oxidative damage and oral disease in exposed populations [1]. Collectively, these findings suggest that salivary ALDHs may contribute to the maintenance of redox homeostasis and epithelial integrity within the oral cavity, while also serving as potential indicators of oral toxicological stress and adaptive mucosal responses.

### 5.3. Salivary ALDH and Local Carcinogen Clearance

The oral cavity represents the first site of exposure to ethanol-derived acetaldehyde generated by resident oral microbiota. Oral epithelial and salivary gland ALDHs contribute to local acetaldehyde detoxification before prolonged epithelial exposure or systemic absorption occurs. Measured salivary ALDH activity likely reflects the capacity of these tissues to metabolize acetaldehyde rather than extensive free enzymatic catalysis within saliva itself. This local detoxification mechanism is particularly important for limiting chronic carcinogenic stress in oral tissues. Individuals with higher salivary ALDH activity often exhibit lower salivary acetaldehyde concentrations, suggesting that salivary ALDH activity may serve as a surrogate marker of effective local aldehyde-detoxification capacity following alcohol consumption, supporting a protective role against aldehyde-mediated oral carcinogenesis [33]. Collectively, these findings position salivary ALDHs, especially ALDH3A1, as central components of oral toxicological defense and important regulators of epithelial resilience within the oral exposome.

## 6. Salivary ALDH in Oral Disease States

Disruption of salivary ALDH activity, whether caused by genetic polymorphisms, chronic oxidative stress, environmental toxicants, inflammation, or metabolic dysfunction, can compromise local aldehyde detoxification and contribute to oral disease progression. Increasing evidence supports the involvement of salivary ALDHs, particularly ALDH3A1 and ALDH1A1, in oral carcinogenesis, periodontal inflammation, mucosal injury, and redox imbalance. Because salivary ALDH activity reflects the dynamic interaction between aldehyde burden and epithelial defense capacity, these enzymes are emerging as potential biomarkers of oral toxicological stress and disease susceptibility. However, interpretation of salivary ALDH activity requires caution. Although reduced salivary ALDH activity is frequently interpreted as impaired aldehyde detoxification capacity, alternative explanations should be considered. Changes in measured activity may also reflect variations in epithelial cell shedding, salivary gland function, inflammatory cell infiltration, extracellular vesicle release, or alterations in enzyme stability within saliva. Therefore, observed associations between salivary ALDH activity and disease states should not necessarily be interpreted as causal without supporting mechanistic evidence. Salivary ALDH activity exhibits a biphasic response in diabetes, with early compensatory upregulation followed by decline in later stages, likely due to glycation-mediated enzyme dysfunction and progressive oxidative stress [1]. Similarly, elevated salivary ALDH1 levels have been reported in erosive and ulcerative oral lichen planus, possibly reflecting an adaptive response to chronic oxidative and inflammatory stress associated with increased malignant potential [38].

### 6.1. Oral Squamous Cell Carcinoma and Precancerous Lesions

Reduced salivary ALDH activity has been associated with increased susceptibility to OSCC and oral potentially malignant disorders. Patients with oral cavity cancer exhibit significantly lower salivary ALDH3A1 activity compared with healthy individuals and patients with non-malignant oral lesions [11]. This reduction may impair detoxification of carcinogenic aldehydes such as acetaldehyde and lipid peroxidation-derived aldehydes, thereby increasing DNA adduct formation, oxidative damage, and epithelial genomic instability.

In early disease stages, ALDH3A1 and ALDH1A1 appear to function protectively by limiting aldehyde-mediated mutagenesis and preserving epithelial redox balance [39]. However, advanced OSCC demonstrates a more complex and isoform-dependent pattern of ALDH regulation. Elevated ALDH activity within tumor tissues, particularly involving ALDH1A1 and ALDH1A3, is associated with cancer stem-like cell populations characterized by enhanced oxidative stress tolerance, metabolic plasticity, and therapeutic resistance [27,40]. These findings suggest that salivary and tissue-associated ALDH activity may shift from a protective detoxification mechanism in early carcinogenesis to a tumor-adaptive survival pathway in advanced disease.

### 6.2. Periodontal Inflammation and Oxidative Stress

Periodontal disease is characterized by chronic inflammation, oxidative stress, and microbial dysbiosis that collectively increase local aldehyde production [41]. Salivary ALDHs contribute to the protection of periodontal tissues by detoxifying reactive aldehydes generated during inflammatory lipid peroxidation and microbial metabolism. This detoxification limits oxidative tissue injury and may help regulate inflammatory signaling within the periodontal microenvironment [42]. Conversely, reduced ALDH activity may exacerbate aldehyde accumulation, oxidative stress, and inflammatory tissue destruction, thereby increasing susceptibility to periodontitis and worsening disease progression. Because salivary ALDH activity reflects local oxidative burden, it may also serve as a non-invasive biomarker for periodontal inflammatory status [43].

### 6.3. Alcohol-Associated Oral Aldehyde Toxicity

The oral cavity is a major site of acetaldehyde generation following alcohol consumption due to microbial and mucosal ethanol metabolism. ALDH enzymes within oral epithelial and salivary gland tissues provide a critical local detoxification barrier by oxidizing acetaldehyde into less toxic acetate. Salivary ALDH activity may therefore serve as a biomarker of this tissue-level detoxification capacity. This protective mechanism becomes particularly important in individuals carrying inactive ALDH2 alleles, who exhibit impaired aldehyde clearance and markedly elevated salivary acetaldehyde concentrations [44]. Chronic aldehyde accumulation under these conditions promotes oxidative DNA damage, epithelial injury, and increased risk of leukoplakia and OSCC, especially when combined with tobacco exposure.

### 6.4. Bidirectional Regulation of Salivary ALDH in Oral Disease

Salivary ALDH activity appears to exhibit context-dependent regulation across oral disease states, reflecting its potential role in adaptation to aldehyde and oxidative stress (Table 1). In inflammatory and early-stage pathological conditions, including diabetes and oral lichen planus, increased salivary ALDH activity has been reported and may represent a compensatory response to elevated oxidative and carbonyl stress [1,38]. Such adaptive upregulation may enhance aldehyde detoxification and help maintain redox homeostasis under conditions of increased cellular stress.

Conversely, reduced salivary ALDH activity has been observed in certain chronic and advanced disease states, including OSCC [11]. However, the mechanisms underlying this decline remain incompletely understood. Rather than indicating a proven failure of the local aldehyde defense system, reduced ALDH activity may reflect altered epithelial metabolism, disease-associated tissue dysfunction, changes in cellular composition, or impaired detoxification capacity. Current evidence is largely cross-sectional and does not establish whether ALDH dysregulation is a cause, consequence, or adaptive response during disease progression. Therefore, the proposed bidirectional regulation of salivary ALDH should be regarded as a conceptual framework for interpreting existing observations rather than a definitive mechanistic model. Future longitudinal and mechanistic studies are needed to clarify the temporal and causal relationships between salivary ALDH activity, aldehyde burden, and oral disease progression.

## 7. Genetic and Environmental Regulation of Salivary ALDH Within the Oral Exposome

**Figure 5:**
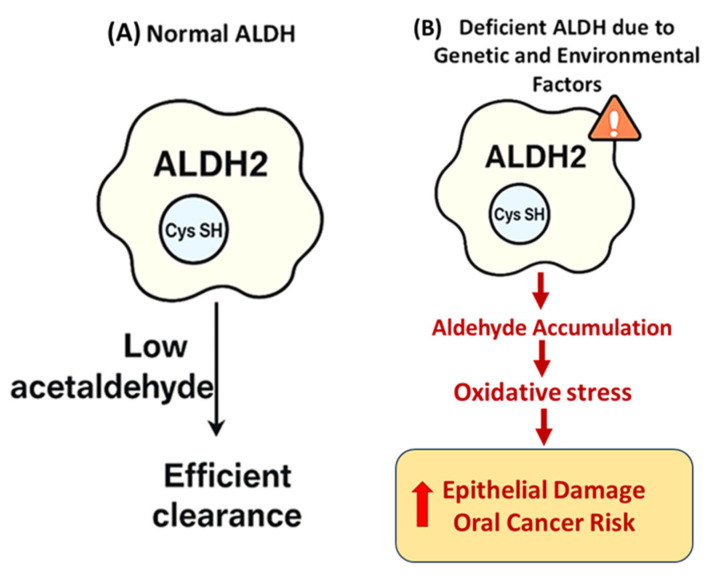
Impact of ALDH2 functionality on acetaldehyde detoxification and oral disease risk. (**A**) Under normal conditions, functional ALDH2 efficiently detoxifies acetaldehyde through its active-site cysteine residue (Cys-SH), resulting in low intracellular acetaldehyde levels and effective clearance. (**B**) Genetic variants or environmental factors that impair ALDH2 activity lead to reduced aldehyde detoxification, causing acetaldehyde accumulation and increased oxidative stress. This imbalance promotes epithelial damage and elevates the risk of oral carcinogenesis.

ALDH3A1 has been proposed as an adaptive responder to oral exposome-associated exposures, although this role remains to be validated in salivary systems (Figure 5). Because ALDH activity depends on intracellular redox balance and NAD(P)^+^ availability, alterations in metabolic and oxidative states further modulate its detoxification efficiency.

### 7.1. Genetic Polymorphisms and Salivary Aldehyde Burden

The ALDH2*2 polymorphism (Glu487Lys/E504K) is a major genetic determinant of impaired aldehyde metabolism and markedly reduced acetaldehyde clearance [46]. Structural disruption of ALDH2 oligomer assembly and catalytic activity results in prolonged aldehyde exposure following alcohol consumption, thereby increasing local and systemic aldehyde burden [47]. Although most mechanistic evidence derives from systemic and hepatic models, these findings are highly relevant to the oral cavity because impaired systemic detoxification likely increases dependence on local salivary ALDH pathways for protection against aldehyde-mediated epithelial injury. Experimental and epidemiological studies consistently associate ALDH2 dysfunction with enhanced oxidative stress, inflammatory signaling, and increased susceptibility to alcohol-associated upper aerodigestive tract cancers [48].

### 7.2. Ethnic Susceptibility and Oral Toxicological Risk

The prevalence of ALDH2 polymorphisms exhibits marked ethnic variation, with ALDH2*2 occurring predominantly in East Asian populations [49]. Individuals carrying this variant exhibit increased salivary acetaldehyde accumulation after alcohol exposure and substantially higher susceptibility to oral and esophageal carcinogenesis, particularly when combined with tobacco use and poor oral hygiene. Experimental studies further demonstrate that alcohol-induced squamous carcinogenesis is markedly enhanced in ALDH2-deficient models, highlighting the synergistic interaction between genetic susceptibility and chronic aldehyde exposure [50]. These observations support the concept that impaired aldehyde detoxification within the oral exposome amplifies epithelial vulnerability to mutagenic and inflammatory stress.

### 7.3. Environmental Regulation of Salivary ALDH: Alcohol, Tobacco, Diet, and Oral Microbiota

Environmental influences on salivary ALDH largely mirror changes occurring within oral epithelial and salivary gland cells. These tissue-associated ALDH systems may be modulated by factors within the oral exposome through effects on oxidative stress, redox homeostasis, inflammatory signaling, and cofactor availability. Alcohol consumption is a major source of aldehyde stress in the oral cavity because ethanol is metabolized locally by oral epithelial cells and acetaldehyde-producing microorganisms, resulting in elevated salivary acetaldehyde concentrations before systemic clearance occurs [34]. Acetaldehyde contributes to DNA adduct formation, protein carbonylation, and lipid peroxidation. Experimental and clinical studies suggest that chronic aldehyde exposure may promote adaptive upregulation of ALDH3A1 in oral epithelial tissues, potentially through oxidative stress-responsive pathways such as Nrf2 signaling [8,51]. Conversely, persistent alcohol-associated oxidative stress has been proposed to impair ALDH function through mechanisms including glycation, oxidative modification, NAD^+^ depletion, mitochondrial dysfunction, and chronic inflammatory signaling. However, these mechanisms are supported primarily by systemic and experimental studies and remain to be validated in salivary tissues.

Tobacco smoke introduces reactive aldehydes, including acetaldehyde, acrolein, formaldehyde, and crotonaldehyde, that contribute to oxidative and genotoxic stress. Smokers exhibit reduced salivary antioxidant capacity and increased oxidative stress markers [52]. Experimental epithelial studies further suggest that chronic tobacco exposure may impair ALDH function through oxidative protein modification, glutathione depletion, altered cofactor availability, and suppression of ALDH3A1 expression, although direct salivary evidence remains limited.

Dietary phytochemicals such as EGCG, resveratrol, curcumin, and quercetin may indirectly influence ALDH-regulated pathways through activation of Nrf2-, SIRT1-, and ARE-dependent antioxidant signaling [53,54]. Evidence from systemic and epithelial models indicates that these compounds reduce aldehyde accumulation and oxidative stress; however, their effects on salivary ALDH activity remain largely unexplored.

The oral microbiota is another important determinant of aldehyde burden because several oral microorganisms convert ethanol into acetaldehyde. Dysbiosis may further increase aldehyde accumulation through inflammation-associated oxidative stress and lipid peroxidation. Adequate ALDH activity within oral tissues may help limit exposure to these aldehydes. A proposed but as yet unvalidated concept is the existence of an ALDH–microbiome feedback loop, whereby impaired ALDH activity may contribute to aldehyde accumulation and microbial dysbiosis, while microbial metabolites and chronic inflammation further influence ALDH function. Although biologically plausible, this model currently remains hypothetical and requires direct experimental validation in salivary and oral tissue systems. Overall, many of the mechanisms discussed above are inferred from epithelial and systemic ALDH studies and should be regarded as biologically plausible models rather than established salivary-specific pathways.

## 8. Salivary ALDH Signaling in Oral Disease: Molecular Mechanisms and Translational Relevance

Beyond their role in aldehyde detoxification, ALDHs participate in broader redox-sensitive signaling networks that support epithelial adaptation and regulate inflammatory responses. By metabolizing reactive aldehydes generated during lipid peroxidation and oxidative stress, ALDH activity helps maintain intracellular redox homeostasis and limits the accumulation of secondary toxic mediators [55]. In particular, Experimental evidence suggests that ALDH3A1 may contribute to cellular antioxidant defenses through NAD(P)^+^-dependent aldehyde oxidation, which supports the maintenance of reducing equivalents and indirectly sustains antioxidant systems such as glutathione and thioredoxin. Experimental studies in epithelial models further indicate that ALDH3A1 attenuates oxidative stress-induced inflammatory signaling and protects against aldehyde-mediated cellular injury. Conversely, ALDH expression itself appears to be regulated by oxidative stress-responsive pathways, most notably the Nrf2–ARE signaling axis [27,56]. Under conditions of increased aldehyde burden, Nrf2 activation enhances the expression of cytoprotective genes, including ALDH family members, thereby strengthening aldehyde detoxification capacity and promoting epithelial stress tolerance. Although direct evidence in salivary tissues remains limited, this reciprocal interaction between ALDH activity and Nrf2 signaling likely represents an important adaptive mechanism for maintaining oral tissue resilience within the oral exposome.

Salivary ALDH activity may additionally influence NAD^+^-dependent signaling pathways involved in cellular stress adaptation. Experimental evidence suggests that preservation of ALDH activity supports intracellular NAD^+^ homeostasis, thereby indirectly modulating SIRT1-mediated signaling networks associated with inflammation control, oxidative stress resistance, and epithelial survival [30,56]. Through these interactions, ALDH-associated redox regulation may suppress NF-κB-dependent inflammatory signaling, enhance FOXO3-mediated antioxidant responses, and support epithelial recovery following oxidative injury. While most mechanistic evidence derives from epithelial and systemic models, these pathways are highly relevant to salivary tissues chronically exposed to aldehyde stress.

Beyond redox regulation, ALDH3A1 also contributes to epithelial stress adaptation and tissue integrity. Under oxidative conditions, ALDH3A1 has been reported to interact with cell cycle regulatory pathways, including p53-associated stress responses, thereby limiting excessive epithelial proliferation and promoting survival under chemical insult [57]. ALDH1A1 additionally contributes to retinoic acid biosynthesis, linking ALDH activity to epithelial differentiation and mucosal repair mechanisms [58]. These isoform-specific functions suggest that salivary ALDHs participate in broader epithelial adaptation programs beyond simple aldehyde detoxification.

Emerging evidence further supports a role for salivary ALDHs in regulating inflammatory and immune responses within the oral microenvironment. Reactive aldehydes generated during oxidative stress can function as danger-associated molecular patterns (DAMPs), activating inflammatory pathways including RAGE and TLR4 signaling [59]. By reducing aldehyde accumulation, salivary ALDHs may help suppress persistent inflammatory activation and limit oxidative tissue injury. Inferred from broader ALDH biology, ALDH-mediated maintenance of redox balance may additionally influence cytokine production, epithelial–immune communication, and inflammatory resolution within chronically stressed oral tissues.

Salivary ALDH signaling is also closely linked to epithelial regeneration and tissue resilience. In radiation-induced salivary gland injury, ALDH3A1 protects salivary stem/progenitor cells (SSPCs) against aldehyde-induced oxidative damage by detoxifying lipid peroxidation products and preserving mitochondrial integrity [30]. Activation of ALDH3A1 improves SSPC survival, regenerative capacity, and salivary gland recovery following radiation exposure, highlighting its translational relevance in treatment-associated xerostomia and mucosal injury.

In oral carcinogenesis, salivary ALDH signaling demonstrates context-dependent duality. In early inflammatory or precancerous conditions, elevated ALDH expression may represent an adaptive protective response against oxidative DNA damage and aldehyde accumulation [38]. However, in established OSCC, ALDH-high tumor cell populations exhibit enhanced oxidative stress tolerance, clonogenicity, stemness-associated signaling, and therapeutic resistance [60,61]. These observations suggest a transition from protective epithelial detoxification toward tumor-adaptive survival signaling during malignant progression. Understanding this context-dependent shift may improve biomarker interpretation and support the development of isoform-specific therapeutic strategies. Collectively, these findings position salivary ALDHs as dynamic regulators of epithelial resilience, redox adaptation, and inflammatory homeostasis within the oral exposome. Their integration with oxidative stress signaling, epithelial regeneration, and carcinogenic pathways supports emerging translational applications in salivary diagnostics, oral toxicology, radiation injury, and precision therapeutics targeting aldehyde-associated oral disease.

The proposed salivary ALDH functional axis linking aldehyde detoxification, redox regulation, epithelial adaptation, and disease susceptibility should be viewed as a conceptual framework derived from the integration of available evidence rather than a fully established biological pathway. While supported by findings from salivary, epithelial, and broader ALDH studies, validation of this model will require dedicated mechanistic and longitudinal clinical investigations.

## 9. Therapeutic and Preventive Implications of Salivary ALDH

**Figure 6:**
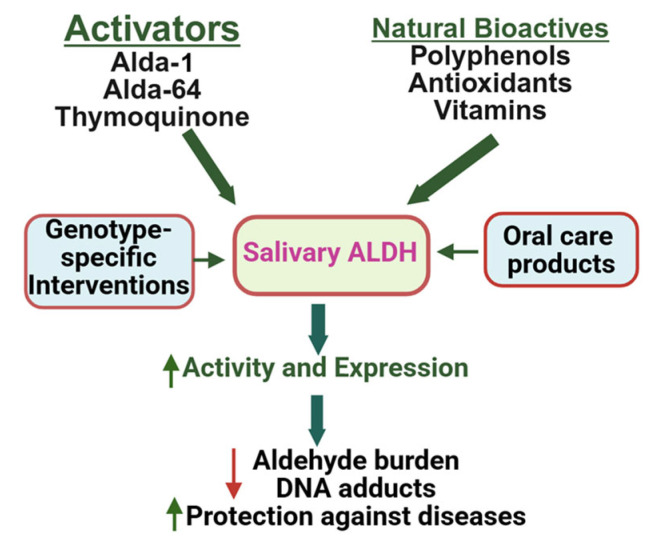
Therapeutic strategies to enhance salivary ALDH activity. Experimental studies support the ability of pharmacological ALDH activators, such as Alda-1 and related compounds, to enhance ALDH activity. Evidence for salivary ALDH modulation by thymoquinone, alliin, and sulforaphane is currently limited to a small number of in vitro studies, primarily from a single research group, and therefore should be considered preliminary pending independent validation. Genotype-specific interventions targeting ALDH polymorphisms and the incorporation of ALDH-modulating compounds into oral care products are presented as proposed or emerging strategies, offering localized protection and accessible delivery formats with translational potential.

Salivary ALDHs, particularly ALDH3A1, are emerging as promising targets for oral toxicology-based therapeutic and preventive strategies because of their central role in aldehyde detoxification, epithelial protection, and redox homeostasis within the oral cavity. Therapeutic modulation of salivary ALDH activity may help reduce acetaldehyde accumulation, suppress oxidative stress, limit aldehyde-induced DNA damage, and preserve epithelial integrity in individuals exposed to chronic aldehyde burden from alcohol, tobacco smoke, inflammation, and microbial metabolism (Figure 6, Table 2). Current evidence supporting ALDH-targeted interventions derives primarily from in vitro studies, epithelial models, and systemic ALDH research, whereas direct salivary clinical evidence remains limited.

Pharmacological activation of ALDH represents one of the most direct approaches for enhancing local aldehyde detoxification capacity. Alda-1, a small-molecule activator of ALDH2, restores catalytic activity in ALDH2*2 variants and improves aldehyde clearance in systemic models [62]. Although its specific effects on salivary ALDH isoforms have not yet been clinically established, these findings provide mechanistic support for localized ALDH-targeted therapies within the oral cavity. Similarly, thymoquinone has been reported to enhance human salivary ALDH activity in an in vitro biochemical study through improved substrate affinity and catalytic efficiency [63]. However, this observation currently derives from a single research group and has not yet been independently replicated in oral tissue, animal, or clinical studies [63]. Changes in salivary ALDH activity may serve as a non-invasive biomarker for monitoring therapeutic efficacy. Alliin has also been reported to improve salivary ALDH kinetics through increased catalytic efficiency in vitro [64]. However, evidence remains limited to a single mechanistic study, and independent confirmation of these findings is currently lacking [64]. Collectively, these preliminary findings suggest that selective modulation of salivary ALDH activity may represent a potential future strategy for reducing oral aldehyde burden. However, the available evidence remains largely exploratory and requires independent validation before therapeutic applicability can be established.

Natural compounds and dietary phytochemicals may additionally preserve salivary ALDH function through indirect redox-regulatory mechanisms. For salivary ALDH specifically, direct evidence is currently limited. The reported activation of human salivary ALDH by sulforaphane derives from an in vitro study and has not yet undergone independent replication or clinical validation [52,65,66]. These compounds reduce reactive aldehyde accumulation, suppress lipid peroxidation, and attenuate glyco-oxidative stress, mechanisms that may indirectly stabilize salivary ALDH activity under chronic oxidative conditions. However, most of these mechanisms remain inferred from non-salivary models, and direct clinical evidence for preservation of salivary ALDH function is currently lacking. In addition, bioavailability limitations, rapid oral clearance, and variability in local uptake remain important translational challenges for oral delivery of natural compounds.

Localized therapeutic delivery systems represent another promising but largely hypothetical area of translational development. Conceptually, incorporation of ALDH-enhancing compounds into oral rinses, mucoadhesive gels, lozenges, chewing gums, nanoparticle formulations, or slow-release dental materials could enable prolonged local exposure and targeted modulation of salivary ALDH activity within oral tissues. Such approaches may be particularly relevant for individuals with chronic aldehyde exposure, ALDH2 polymorphisms, radiation-induced salivary dysfunction, or persistent inflammatory oral disease. However, these applications remain largely speculative because no clinical trials have specifically evaluated ALDH-targeted oral formulations for preservation or enhancing oral epithelial and salivary gland ALDH activity.

Salivary ALDHs also have potential translational value as biomarkers for early risk assessment and personalized intervention. Individuals carrying ALDH2*2 polymorphisms exhibit increased aldehyde accumulation and higher susceptibility to alcohol-associated oral carcinogenesis [44]. Measurement of salivary ALDH activity may therefore provide a non-invasive indicator of aldehyde detoxification capacity, epithelial oxidative stress, and oral toxicological vulnerability. In high-risk populations with chronic alcohol or tobacco exposure, longitudinal monitoring of salivary ALDH activity could potentially support individualized preventive strategies, including lifestyle modification, aldehyde exposure reduction, and targeted antioxidant interventions.

Therapeutic modulation of salivary ALDH activity must also consider disease context and isoform-specific effects. The therapeutic targeting of ALDHs presents important challenges. While enhancement of ALDH activity may improve aldehyde detoxification and epithelial resilience in certain contexts, excessive or persistent ALDH expression has been associated with tumor cell survival, stemness, and therapeutic resistance in several cancers. Consequently, the biological effects of ALDH modulation are likely to be context- and isoform-dependent. Therapeutic strategies aimed at enhancing ALDH activity may therefore require careful patient stratification and disease-specific application.

In early inflammatory and precancerous conditions, enhancing oral epithelial and salivary gland ALDH activity may strengthen epithelial detoxification and reduce aldehyde-mediated DNA damage. In contrast, advanced oral squamous cell carcinoma may contain ALDH-high tumor cell populations associated with stemness, therapeutic resistance, and survival signaling [44]. Consequently, indiscriminate ALDH activation may not always be beneficial in established malignancy, highlighting the importance of context-specific and isoform-selective therapeutic approaches. Future development of salivary ALDH-targeted therapies will therefore require improved understanding of isoform biology, local delivery systems, epithelial redox regulation, and disease-stage-dependent ALDH signaling within the oral exposome.

## 10. Future Perspectives and Research Directions

Despite growing interest in salivary ALDHs as diagnostic and therapeutic targets, their clinical utility remains uncertain. Current evidence is derived largely from in vitro studies, animal models, and mechanistic extrapolations from systemic ALDH biology, with relatively few saliva-specific investigations and limited independent replication of reported salivary ALDH modulators. For example, compounds such as sulforaphane, thymoquinone, and alliin have been reported to enhance salivary ALDH activity, but these findings require confirmation in independent preclinical and clinical studies before therapeutic relevance can be established.

Several factors also limit the diagnostic application of salivary ALDHs. Measured activity is influenced by age, smoking, alcohol consumption, oral microbiota composition, oral hygiene, inflammatory status, and genetic background, reducing disease specificity. In addition, most current assays quantify total ALDH activity and lack the isoform specificity needed to distinguish the contributions of ALDH3A1, ALDH1A1, and other family members. The absence of standardized analytical methods, validated reference ranges, and longitudinal clinical data further constrains interpretation and clinical implementation. Collectively, these limitations highlight the need for rigorous saliva-specific mechanistic studies, assay standardization, prospective clinical validation, and well-designed intervention trials before salivary ALDHs can be reliably incorporated into precision oral healthcare.

Methodological standardization represents another major priority. Most current assays rely on nonspecific spectrophotometric or fluorometric detection of NAD(P)H generation and cannot reliably distinguish among individual ALDH isoforms. Future efforts should focus on developing sensitive isoform-specific assays, biosensors, and microfluidic point-of-care platforms capable of quantifying salivary ALDH activity with improved specificity. Establishment of population-based reference ranges stratified by age, sex, environmental exposures, and ALDH genotype would further enhance clinical applicability.

Large longitudinal studies are also needed to determine whether altered salivary ALDH activity represents a causal factor, adaptive response, or consequence of disease progression. Prospective investigations in individuals with oral potentially malignant disorders, periodontitis, radiation-induced salivary dysfunction, metabolic disease, and chronic alcohol or tobacco exposure may help clarify the prognostic value of salivary ALDH measurements. Important mechanistic questions likewise remain unresolved. While ALDH3A1 and ALDH1A1 are increasingly implicated in oral aldehyde metabolism and epithelial homeostasis, upstream and downstream signaling interactions, post-translational regulation, isoform-specific substrate selectivity, and kinetic properties remain poorly characterized in salivary systems. Advanced proteomic and structural approaches, including redox proteomics, glycation-site mapping, and mass spectrometry-based analyses, combined with salivary gland and oral organoid models, may provide important insights into the regulation of salivary ALDHs under physiological and pathological conditions.

Future studies should also investigate emerging concepts such as the proposed salivary ALDH functional axis and the hypothesized ALDH–microbiome feedback loop. Integration of microbiome profiling, metabolomics, aldehyde measurements, and salivary ALDH assessment may improve understanding of how microbial dysbiosis, aldehyde burden, and epithelial stress responses interact within the oral exposome. Finally, integration of salivary ALDH research with multi-omics technologies offers new opportunities for precision oral medicine. Genomic, transcriptomic, proteomic, and metabolomic approaches may help identify exposure-responsive pathways, characterize isoform-specific regulation, and improve understanding of aldehyde-associated disease susceptibility. Together, these advances may facilitate the development of more personalized strategies for risk assessment, prevention, and therapeutic intervention.

## 11. Conclusions

Salivary ALDHs represent an emerging area of research at the intersection of oral toxicology, redox biology, and oral medicine. Current evidence indicates that measurable salivary ALDH activity primarily reflects intracellular and vesicle-associated enzymes originating from oral epithelial cells, salivary gland tissues, and immune cells, and should therefore be interpreted as an indicator of tissue-level aldehyde detoxification capacity and redox status rather than freely functioning extracellular catalysis.

This review highlights the oral exposome as a useful framework for understanding how environmental and endogenous stressors collectively influence aldehyde burden within the oral cavity. Within this context, ALDH enzymes expressed in oral tissues may contribute to aldehyde detoxification, epithelial adaptation, and maintenance of mucosal homeostasis. Among the salivary isoforms, ALDH3A1 has emerged as a potentially important mediator of these processes, although direct saliva-specific mechanistic evidence remains limited. Altered salivary ALDH activity has been reported in association with several oral and systemic conditions, suggesting potential utility as a non-invasive indicator of aldehyde-associated stress. However, many proposed mechanisms, including the ALDH–microbiome feedback loop and the broader salivary ALDH functional axis, remain hypothetical and require dedicated validation in salivary models and clinical studies.

Although pharmacological, nutraceutical, and oral care-based approaches aimed at modulating ALDH activity have shown encouraging results in experimental settings, their translational relevance remains uncertain. Importantly, salivary ALDHs should not currently be regarded as validated clinical biomarkers or therapeutic targets. Their clinical utility remains constrained by limited saliva-specific mechanistic evidence, methodological variability, and a lack of prospective human validation. Nevertheless, the available evidence supports continued investigation of salivary ALDHs as potentially informative indicators of oral exposome-associated stress and as promising subjects for future translational research.

## Figures and Tables

**Table 1:** Bidirectional regulation of salivary ALDH in selected disease states.

Condition	Salivary ALDH Activity	Evidence-Supported Interpretation	Evidence Level	Ref.
Oral Lichen Planus	Upregulated	Elevated salivary ALDH1 levels may represent an adaptive response to increased oxidative stress and may be associated with lesions exhibiting higher malignant potential.	Direct salivary evidence	[38]
Type 2 Diabetes	Upregulated (early), Downregulated (advanced disease)	Early elevation likely reflects a compensatory response to oxidative stress; subsequent decline is associated with increased oxidative/glyco-oxidative stress, although the precise mechanism of ALDH suppression remains unresolved.	Direct salivary evidence with mechanistic inference	[45]
Periodontal Inflammation	Upregulated	Increased oxidative stress may induce ALDH-mediated aldehyde detoxification and redox adaptation; direct isoform-specific salivary evidence remains limited.	Predominantly mechanistic inference	[43]
Oral Squamous Cell Carcinoma (OSCC)	Reduced salivary activity	Reduced salivary ALDH activity has been reported in OSCC patients and may reflect impaired aldehyde detoxification capacity. Associations with ALDH1A1/ALDH3A1 expression and cancer stemness are derived primarily from tumor tissue studies rather than saliva.	Mixed salivary and tissue evidence	[40,44]
Radiation-Induced Salivary Gland Damage	Downregulated	Aldehyde accumulation and oxidative stress impair salivary gland function; activation of ALDH3A1 preserves salivary stem/progenitor cell survival and gland regeneration.	Experimental tissue evidence	[30]

**Table 2:** Therapeutic and Preventive Strategies Targeting Salivary ALDH.

Strategy	Mechanism	Example/Application	Evidence Level
Pharmacological activation	Upregulate ALDH function	Alda-1 and Alda-64	Preclinical (multiple independent studies)
	Enhance salivary ALDH kinetics	Thymoquinone	Single in vitro study
		Alliin	
Dietary Modulation	Nrf2 activation	Sulforaphane,	Single salivary ALDH in vitro study
	Antioxidant signaling	Curcumin, EGCG, Resveratrol	Indirect evidence from non-salivary models
Oral Hygiene Integration	Deliver ALDH activators or cofactors topically	Mouthwash with polyphenols, ALDH-enhancing toothpaste	Conceptual
Functional Foods	Provide sustained release of ALDH-supportive compounds	Herbal lozenges, probiotic beverages	Preclinical/Indirect Evidence
Personalized Screening	Identify at-risk individuals through salivary ALDH + genotyping	Point-of-care biosensors, SNP analysis	Emerging Clinical Evidence
Adjunct Therapy in Disease	Reduce oxidative stress, inflammation, and DNA damage	Periodontal gels, anticancer adjuncts	Experimental/Early Translational

## Data Availability

No data sharing statement is applicable for this article, as it does not involve the generation of new data.

## References

[1] Younus H., Arsalan A., Alam M.F. (2020). Arsenic inhibits human salivary aldehyde dehydrogenase: Mechanism and a population-based study. Chemosphere.

[2] Xanthis V., Mantso T., Dimtsi A., Pappa A., Fadouloglou V.E. (2023). Human Aldehyde Dehydrogenases: A Superfamily of Similar Yet Different Proteins Highly Related to Cancer. Cancers.

[3] Singh S., Brocker C., Koppaka V., Chen Y., Jackson B.C., Matsumoto A., Thompson D.C., Vasiliou V. (2013). Aldehyde dehydrogenases in cellular responses to oxidative/electrophilic stress. Free Radic. Biol. Med..

[4] Dumitru C.N., Mariana L., Budacu C.C., Mitea G., Radu M.D., Dumitru A.O., Lupoae A., Tatu A., Topor G. (2025). Balancing the Oral Redox State: Endogenous and Exogenous Sources of Reactive Oxygen Species and the Antioxidant Role of Lamiaceae and Asteraceae. Dent. J..

[5] Stornetta A., Guidolin V., Balbo S. (2018). Alcohol-Derived Acetaldehyde Exposure in the Oral Cavity. Cancers.

[6] Sapkota M., Wyatt T.A. (2015). Alcohol, Aldehydes, Adducts and Airways. Biomolecules.

[7] Jang J.H., Bruse S., Liu Y., Duffy V., Zhang C., Oyamada N., Randell S., Matsumoto A., Thompson D.C., Lin Y. (2014). Aldehyde dehydrogenase 3A1 protects airway epithelial cells from cigarette smoke-induced DNA damage and cytotoxicity. Free Radic. Biol. Med..

[8] Liu T., Zhang F., Feng Y., Han P., Gao Y. (2025). Alcohol-Metabolizing Enzymes, Liver Diseases and Cancer. Semin. Liver Dis..

[9] Nieminen M.T., Salaspuro M. (2018). Local Acetaldehyde-An Essential Role in Alcohol-Related Upper Gastrointestinal Tract Carcinogenesis. Cancers.

[10] Ng C.S., Ong X.J., Au M., Lau Y.H., Kwok H.H.Y., Quan J. (2023). ALDH2 polymorphism, alcohol intake and the attributable burden of cancer in East Asia: Systematic review, meta-analysis, and modeling study. Ann. Epidemiol..

[11] Giebułtowicz J., Wroczyński P., Samolczyk-Wanyura D. (2013). Can lower aldehyde dehydrogenase activity in saliva be a risk factor for oral cavity cancer?. Oral Dis..

[12] Abraham D., Gupta A., Duraisamy A.K., Mrinalini M. (2025). The influence of chronic periodontitis and type 2 diabetes mellitus on resistin levels of gingival crevicular fluid- a systematic review and meta-analysis. J. Oral Biol. Craniofac. Res..

[13] Belstrøm D. (2020). The salivary microbiota in health and disease. J. Oral Microbiol..

[14] International Agency for Research on Cancer (2012). IARC Monographs on the Evaluation of Carcinogenic Risks to Humans; A Review of Human Carcinogens.

[15] Duan X., Hu H., Wang L., Chen L. (2024). Aldehyde dehydrogenase 1 family: A potential molecule target for diseases. Cell Biol. Int..

[16] Chen C.H., Joshi A.U., Mochly-Rosen D. (2016). The Role of Mitochondrial Aldehyde Dehydrogenase 2 (ALDH2) in Neuropathology and Neurodegeneration. Acta Neurol. Taiwan.

[17] Magrassi L., Pinton G., Luzzi S., Comincini S., Scravaglieri A., Gigliotti V., Bernardoni B.L., D’Agostino I., Juretich F., La Motta C. (2024). A New Vista of Aldehyde Dehydrogenase 1A3 (ALDH1A3): New Specific Inhibitors and Activity-Based Probes Targeting ALDH1A3 Dependent Pathways in Glioblastoma, Mesothelioma and Other Cancers. Cancers.

[18] Saiki J.P., Cao H., Van Wassenhove L.D., Viswanathan V., Bloomstein J., Nambiar D.K., Mattingly A.J., Jiang D., Chen C.H., Stevens M.C. (2018). Aldehyde dehydrogenase 3A1 activation prevents radiation-induced xerostomia by protecting salivary stem cells from toxic aldehydes. Proc. Natl. Acad. Sci. USA.

[19] Shortall K., Djeghader A., Magner E., Soulimane T. (2021). Insights into Aldehyde Dehydrogenase Enzymes: A Structural Perspective. Front. Mol. Biosci..

[20] Poturnajova M., Kozovska Z., Matuskova M. (2021). Aldehyde dehydrogenase 1A1 and 1A3 isoforms—Mechanism of activation and regulation in cancer. Cell Signal.

[21] Zhao W., Xia Y., Gao Z., Chen J., Zhang E. (2025). Multiple roles of ALDH1 in health and disease. Front. Physiol..

[22] Clark D.W., Palle K. (2016). Aldehyde dehydrogenases in cancer stem cells: Potential as therapeutic targets. Ann. Transl. Med..

[23] Hrelia P., Sita G., Ziche M., Ristori E., Marino A., Cordaro M., Molteni R., Spero V., Malaguti M., Morroni F. (2020). Common Protective Strategies in Neurodegenerative Disease: Focusing on Risk Factors to Target the Cellular Redox System. Oxid. Med. Cell Longev..

[24] Chen C.H., Ferreira J.C., Gross E.R., Mochly-Rosen D. (2014). Targeting aldehyde dehydrogenase 2: New therapeutic opportunities. Physiol. Rev..

[25] Wang Q., Chang B., Li X., Zou Z. (2021). Role of ALDH2 in Hepatic Disorders: Gene Polymorphism and Disease Pathogenesis. J. Clin. Transl. Hepatol..

[26] Swain N., Thakur M., Pathak J., Patel S., Hosalkar R. (2022). Aldehyde dehydrogenase 1: Its key role in cell physiology and oral carcinogenesis. Dent. Med. Probl..

[27] Rodriguez-Torres M., Allan A.L. (2016). Aldehyde dehydrogenase as a marker and functional mediator of metastasis in solid tumors. Clin. Exp. Metastasis.

[28] Zhang J., Cai B., Ma M., Luo W., Zhang Z., Zhang X., Nie Q. (2020). ALDH1A1 Inhibits Chicken Preadipocytes’ Proliferation and Differentiation via the PPARγ Pathway In Vitro and In Vivo. Int. J. Mol. Sci..

[29] Voulgaridou G.P., Tsochantaridis I., Tolkas C., Franco R., Giatromanolaki A., Panayiotidis M.I., Pappa A. (2020). Aldehyde dehydrogenase 3A1 confers oxidative stress resistance accompanied by altered DNA damage response in human corneal epithelial cells. Free Radic. Biol. Med..

[30] Viswanathan V., Cao H., Saiki J., Jiang D., Mattingly A., Nambiar D., Bloomstein J., Li Y., Jiang S., Chamoli M. (2022). Aldehyde dehydrogenase 3A1 deficiency leads to mitochondrial dysfunction and impacts salivary gland stem cell phenotype. PNAS Nexus.

[31] Schwartz M., Neiers F., Charles J.P., Heydel J.M., Muñoz-González C., Feron G., Canon F. (2021). Oral enzymatic detoxification system: Insights obtained from proteome analysis to understand its potential impact on aroma metabolization. Compr. Rev. Food Sci. Food Saf..

[32] Li Pomi F., Gammeri L., Borgia F., Di Gioacchino M., Gangemi S. (2025). Oxidative Stress and Skin Diseases: The Role of Lipid Peroxidation. Antioxidants.

[33] Zhang Y., Wang M., Lin H. (2020). A Regulatory Cysteine Residue Mediates Reversible Inactivation of NAD^+^-Dependent Aldehyde Dehydrogenases to Promote Oxidative Stress Response. ACS Chem. Biol..

[34] Thapa M.J., Chan K. (2025). The mutagenic properties of formaldehyde and acetaldehyde: Reflections on half a century of progress. Mutat. Res..

[35] Jin S., Chen J., Chen L., Histen G., Lin Z., Gross S., Hixon J., Chen Y., Kung C., Chen Y. (2015). ALDH2(E487K) mutation increases protein turnover and promotes murine hepatocarcinogenesis. Proc. Natl. Acad. Sci. USA.

[36] Li S., Tan H.-Y., Wang N., Zhang Z.-J., Lao L., Wong C.-W., Feng Y. (2015). The Role of Oxidative Stress and Antioxidants in Liver Diseases. Int. J. Mol. Sci..

[37] Zhang X., Hou L., Guo Z., Wang G., Xu J., Zheng Z., Sun K., Guo F. (2023). Lipid peroxidation in osteoarthritis: Focusing on 4-hydroxynonenal, malondialdehyde, and ferroptosis. Cell Death Discov..

[38] Mansourian A., Shanbehzadeh N., Kia S.J., Moosavi M.S. (2017). Increased salivary aldehyde dehydrogenase 1 in non-reticular oral lichen planus. An. Bras. Dermatol..

[39] Eloranta R., Vilén S.T., Keinänen A., Salo T., Qannam A., Bello I.O., Snäll J. (2024). Oral squamous cell carcinoma: Effect of tobacco and alcohol on cancer location. Tob. Induc. Dis..

[40] Fatima Khan R., Akbar Ansari S., Bukhari U., Taqi M., Farooqi S., Ali Muhammad A. (2025). Association of ALDH3A1 expression with tumor differentiation, pathological stage, and nodal status in oral squamous cell carcinoma. J. Taibah Univ. Med. Sci..

[41] Shang J., Liu H., Zheng Y., Zhang Z. (2023). Role of oxidative stress in the relationship between periodontitis and systemic diseases. Front. Physiol..

[42] Tsai H.C., Chen C.H., Mochly-Rosen D., Li Y.E., Chen M.H. (2021). The Role of Alcohol, LPS Toxicity, and ALDH2 in Dental Bony Defects. Biomolecules.

[43] Wang J., Schipper H.M., Velly A.M., Mohit S., Gornitsky M. (2015). Salivary biomarkers of oxidative stress: A critical review. Free Radic. Biol. Med..

[44] Chang J.S., Hsiao J.R., Chen C.H. (2017). ALDH2 polymorphism and alcohol-related cancers in Asians: A public health perspective. J. Biomed. Sci..

[45] Younus H., Ahmad S., Alam M.F. (2020). Correlation between the Activity of Aldehyde Dehydrogenase and Oxidative Stress Markers in the Saliva of Diabetic Patients. Protein Pept. Lett..

[46] Chang Y.C., Lee H.L., Yang W., Hsieh M.-L., Liu C.-C., Lee T.-Y., Huang J.-Y., Nong J.-Y., Li F.-A., Chuang H.-L. (2023). A common East-Asian ALDH2 mutation causes metabolic disorders and the therapeutic effect of ALDH2 activators. Nat. Commun..

[47] Rwere F., White J.R., Hell R.C.R., Yu X., Zeng X., McNeil L., Zhou K.N., Angst M.S., Chen C.-H., Mochly-Rosen D. (2024). Uncovering newly identified aldehyde dehydrogenase 2 genetic variants that lead to acetaldehyde accumulation after an alcohol challenge. J. Transl. Med..

[48] Okura T., Nakamura R., Anno M., Ito Y., Kitao S., Endo S., Taneda N., Matsumoto K., Shoji K., Okura H. (2023). Aldehyde dehydrogenase 2 polymorphism is an important gene for insulin resistance in Japanese patients with type 2 diabetes. Metab. Open.

[49] Zhong Z., Hou J., Li B., Zhang Q., Li C., Liu Z., Yang M., Zhong W., Zhao P. (2018). Genetic Polymorphisms of the Mitochondrial Aldehyde Dehydrogenase ALDH2 Gene in a Large Ethnic Hakka Population in Southern China. Med. Sci. Monit..

[50] Kondo Y., Ohashi S., Katada C., Nakai Y., Yamamoto Y., Tamaoki M., Kikuchi O., Yamada A., Hirohashi K., Mitani Y. (2025). Aldh2 and the tumor suppressor Trp53 play important roles in alcohol-induced squamous field cancerization. J. Gastroenterol..

[51] Seitz H.K., Becker P. (2007). Alcohol metabolism and cancer risk. Alcohol Res. Health.

[52] Bakhtiari S., Azimi S., Mehdipour M., Amini S., Elmi Z., Namazi Z. (2015). Effect of Cigarette Smoke on Salivary Total Antioxidant Capacity. J. Dent. Res. Dent. Clin. Dent. Prospect. Fall..

[53] Alam M.F., Laskar A.A., Maryam L., Younus H. (2016). Activation of Human Salivary Aldehyde Dehydrogenase by Sulforaphane: Mechanism and Significance. PLoS ONE.

[54] Luo G., Huang B., Qiu X., Xiao L., Wang N., Gao Q., Yang W., Hao L. (2017). Resveratrol attenuates excessive ethanol exposure induced insulin resistance in rats via improving NAD^+^/NADH ratio. Mol. Nutr. Food Res..

[55] Missihoun T.D., Kotchoni S.O., Bartels D. (2018). Aldehyde Dehydrogenases Function in the Homeostasis of Pyridine Nucleotides in *Arabidopsis thaliana*. Sci. Rep..

[56] Yang K., Yang Y., Long T., Wang X., Chen Y., He C., Li L., Yang X., Jiang M., Hu Y. (2025). Hyperhomocysteinaemia aggravates periodontitis by suppressing the Nrf2/HO-1 signalling pathway. Redox Rep..

[57] Ren R., Wang Z., Wu M., Wang H. (2020). Emerging Roles of SIRT1 in Alcoholic Liver Disease. Int. J. Biol. Sci..

[58] Voulgaridou G.-P., Theologidis V., Venetikidou M., Tsochantaridis I., Tsolou A., Koffa M., Panayiotidis M.I., Pappa A. (2023). Investigating the Functional Roles of Aldehyde Dehydrogenase 3A1 in Human Corneal Epithelial Cells. Int. J. Mol. Sci..

[59] Roh J.S., Sohn D.H. (2018). Damage-Associated Molecular Patterns in Inflammatory Diseases. Immune Netw..

[60] Zanoni M., Bravaccini S., Fabbri F., Arienti C. (2022). Emerging Roles of Aldehyde Dehydrogenase Isoforms in Anti-cancer Therapy Resistance. Front. Med..

[61] Chu X., Tian W., Ning J., Xiao G., Zhou Y., Wang Z., Zhai Z., Tanzhu G., Yang J., Zhou R. (2024). Cancer stem cells: Advances in knowledge and implications for cancer therapy. Signal Transduct. Target Ther..

[62] Perez-Miller S., Younus H., Vanam R., Chen C.H., Mochly-Rosen D., Hurley T.D. (2010). Alda-1 is an agonist and chemical chaperone for the common human aldehyde dehydrogenase 2 variant. Nat. Struct. Mol. Biol..

[63] Laskar A.A., Khan M.A., Askari F., Younus H. (2017). Thymoquinone binds and activates human salivary aldehyde dehydrogenase: Potential therapy for the mitigation of aldehyde toxicity and maintenance of oral health. Int. J. Biol. Macromol..

[64] Laskar A.A., Danishuddin, Khan S.H., Subbarao N., Younus H. (2019). Enhancement in the Catalytic Activity of Human Salivary Aldehyde Dehydrogenase by Alliin from Garlic: Implications in Aldehyde Toxicity and Oral Health. Curr. Pharm. Biotechnol..

[65] Cascajosa-Lira A., Prieto A.I., Pichardo S., Jos A., Cameán A.M. (2024). Protective effects of sulforaphane against toxic substances and contaminants: A systematic review. Phytomedicine.

[66] Ruhee R.T., Suzuki K. (2020). The Integrative Role of Sulforaphane in Preventing Inflammation, Oxidative Stress and Fatigue: A Review of a Potential Protective Phytochemical. Antioxidants.

